# Structural-Functional Characterization and Physiological Significance of Ferredoxin-NADP^+^ Reductase from *Xanthomonas axonopodis* pv. *citri*

**DOI:** 10.1371/journal.pone.0027124

**Published:** 2011-11-09

**Authors:** María Laura Tondo, Matías A. Musumeci, María Laura Delprato, Eduardo A. Ceccarelli, Elena G. Orellano

**Affiliations:** Molecular Biology Division, Instituto de Biología Molecular y Celular de Rosario (IBR), CONICET, Facultad de Ciencias Bioquímicas y Farmacéuticas, Universidad Nacional de Rosario, Rosario, Argentina; Griffith University, Australia

## Abstract

*Xanthomonas axonopodis* pv. citri is a phytopathogen bacterium that causes severe citrus canker disease. Similar to other phytopathogens, after infection by this bacterium, plants trigger a defense mechanism that produces reactive oxygen species. Ferredoxin-NADP^+^ reductases (FNRs) are redox flavoenzymes that participate in several metabolic functions, including the response to reactive oxygen species. *Xanthomonas axonopodis* pv. citri has a gene (*fpr*) that encodes for a FNR (*Xac*-FNR) that belongs to the subclass I bacterial FNRs. The aim of this work was to search for the physiological role of this enzyme and to characterize its structural and functional properties. The functionality of *Xac*-FNR was tested by cross-complementation of a FNR knockout *Escherichia coli* strain, which exhibit high susceptibility to agents that produce an abnormal accumulation of ^•^O_2_^-^. *Xac*-FNR was able to substitute for the FNR in *E. coli* in its antioxidant role. The expression of *fpr* in *X. axonopodis* pv. citri was assessed using semiquantitative RT-PCR and Western blot analysis. A 2.2-fold induction was observed in the presence of the superoxide-generating agents methyl viologen and 2,3-dimethoxy-1,4-naphthoquinone. Structural and functional studies showed that *Xac*-FNR displayed different functional features from other subclass I bacterial FNRs. Our analyses suggest that these differences may be due to the unusual carboxy-terminal region. We propose a further classification of subclass I bacterial FNRs, which is useful to determine the nature of their ferredoxin redox partners. Using sequence analysis, we identified a ferredoxin (XAC1762) as a potential substrate of *Xac*-FNR. The purified ferredoxin protein displayed the typical broad UV-visible spectrum of [4Fe-4S] clusters and was able to function as substrate of *Xac*-FNR in the cytochrome *c* reductase activity. Our results suggest that *Xac*-FNR is involved in the oxidative stress response of *Xanthomonas axonopodis* pv. citri and performs its biological function most likely through the interaction with ferredoxin XAC1762.

## Introduction

*Xanthomonas axonopodis* pv. citri is a Gram-negative obligate aerobic bacterium that is responsible for severe citrus canker disease, which affects most commercial citrus cultivars. The disease appears as raised necrotic corky lesions on the leaves, stems and fruits, which reduces the fruit quality and quantity. The pathogen enters host plant tissues through the stomata or tissue wounds, and the infection is visualized as circular spots on the surface of the leaves. Subsequently, the bacteria colonize the apoplast and cause the leaf epidermis to break due to cell hyperplasia [1], [2].

In response to pathogens, the plant metabolism changes to produce reactive oxygen species, including superoxide radicals (^•^O_2_^-^), hydrogen peroxide (H_2_O_2_), and hydroxyl radicals (•OH) which kill the infectious agent [3]. Therefore, pathogens need to prevent and overcome oxidative stress in order to establish and maintain infections [4]. Different studies have demonstrated the protective role that catalases and peroxidases perform in *Xanthomonas spp* during oxidative stress developed by the plant's defence mechanisms [5], [6]. However, in other Gram-negative bacteria, such as *Escherichia coli* and *Pseudomonas putida,* alternative mechanisms of response to oxidative stress have been reported where ferredoxin-NADP^+^ reductase (FNR) performs an important function [7]–[12].

**Figure 1:**
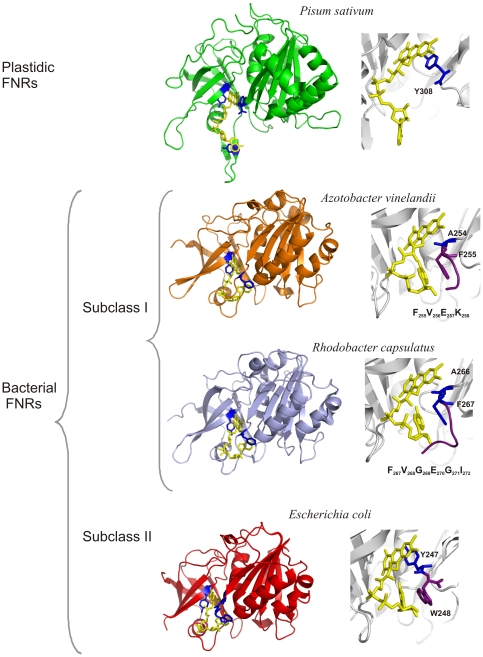
Classification of plant-type FNRs according to structural features. Plant-type FNRs are classified as plastidic and bacterial FNRs [21]. Structures of representative prototypes of the plastidic and the bacterial groups are shown. Bacterial-type FNRs are subdivided into two subclasses, subclass I and subclass II. FNRs from *Azobacter vinelandii* and *Rhodobacter capsulatus* belong to subclass I; however, they differ in length and sequence of the carboxy-terminal region upstream of the alanine that faces the isoalloxazine of FAD. A view of the environments of the different prosthetic groups and the sequences of the carboxy-terminal extensions are shown to the right of each enzyme structure. FNRs from *Pisum sativum* (1qg0), *E. coli* (1fdr), *A. vinelandii* (1a8p) and *R. capsulatus* (2bgj) were used as model proteins. Figures were generated using PyMol. Available: http://pymol.sourceforge.net/.

FNR is a flavoenzyme that is distributed in a large range of organisms. It participates in metabolic processes as dissimilar as photosynthesis [13], nitrogen assimilation [14], [15] and fatty acid desaturation [16]. The FAD prosthetic group enables these enzymes to catalyze electron transfer from obligate two-electron carriers, such as NADP(H), to one-electron proteins [17], such as ferredoxin, flavodoxin [18] or hemoxygenase [19]. In photosyntetic tissues and organisms, the reaction is directed to NADP^+^ reduction to produce NADPH; however, in non-photosynthetic tissues or organisms, the reaction is mainly displaced towards the oxidation of NADPH in order to produce low-potential electron donors that will be used in different metabolic functions [17], [20]. FNRs are grouped into two classes according to their structural and phylogenetic features [21], [22]: a plastidic class, which is found in photosynthetic tissues and displays high catalytic efficiency, and a bacterial class, which has a low catalytic efficiency (Figure 1). The bacterial class is further subdivided into two subclasses: subclass I, which has a structural prototype that is similar to the FNR from *Azotobacter vinelandii,* and subclass II, which is represented by the FNR from *Escherichia coli* [21]. As shown in Figure 1, the main differences between subclasses I and II bacterial FNRs are located in the carboxy-terminal region where *E. coli* FNR (*Ec*-FNR) has a tyrosine that faces the isoalloxazine of FAD, and subclass I enzymes have an alanine in the equivalent position and a longer carboxy-terminal extension [21].

In a previous study, we identified a FNR in *X. axonopodis* pv. citri (*Xac*-FNR) that has all the structural and functional features of a typical subclass I bacterial FNR [23]. The aim of this work was to search for the physiological role of the *Xac*-FNR and its natural substrate in *X. axonopodis* pv. citri and to investigate its participation in the bacterial oxidative stress response. Furthermore, we performed a structural and functional characterization of *Xac*-FNR and analyzed the obtained data in the context of other plant-type FNRs.

## Results and Discussion

### Complementation of an E. coli fpr-null mutant with Xac-FNR

**Figure 2:**
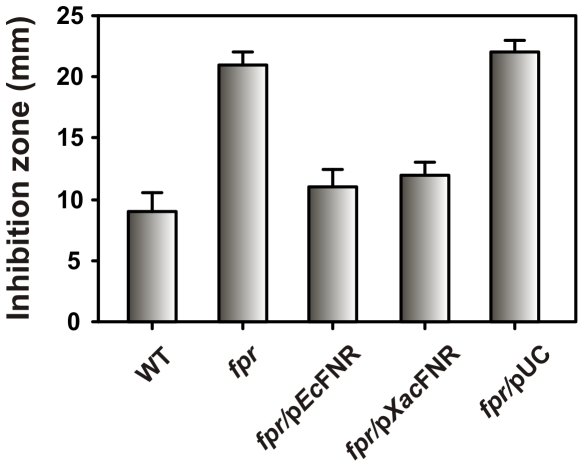
Complementation of the *E. coli fpr*-null mutant with *Xac*-FNR. The *E. coli fpr* strain (RR6A) was transformed with p*Ec*FNR that contained the endogenous *fpr* gene, p*Xac*FNR that contained the *Xac fpr* gene, or pUC119. The susceptibility of *E. coli* strains to MV toxicity was evaluated using the disk diffusion assay. The diameters of the inhibition zones were measured after 24 h of incubation. Bars indicate mean ± standard deviation of three independent experiments.

The functionality of *Xac*-FNR was initially tested by cross-complementation of an *E. coli fpr* mutant (the *fpr* gene encodes *Ec*-FNR). The *E. coli fpr* RR6A strain exhibited a high susceptibility to the bactericidal effects of methyl viologen (MV) [9]. These bacteria displayed a lower growth rate compared to wild-type cells when exposed to oxidants due to the abnormal accumulation of ^•^O_2_^-^ in the cytosol [9]. The *Xac*-FNR coding sequence was amplified and cloned into the pUC119 vector, and the resulting plasmid (pUC/*Xac*FNR) was transformed into *E. coli* RR6A. The resistance to MV of the resulting strain was evaluated using the inhibition zone assay. As shown in Figure 2, the *Xac*-FNR enzyme was able to restore the *E. coli* mutant to similar levels of resistance as the wild-type strain. This result indicates that *Xac*-FNR is able to substitute *Ec*-FNR in its antioxidant role. Attempts to construct a *X. axonopodis* pv. citri *fpr*-knockout strain were unsuccessful. This observation may result from the protective function of *Xac*-FNR or from another role of the enzyme yet to be uncovered. In general, the impossibility to recover knockout strains of a gene in bacteria suggests its participation in essential housekeeping metabolic steps. This issue needs to be further investigated.

The role of FNRs in oxidative stress protection is not completely understood. One of the possible targets of superoxide toxicity are the metal-dependent hydro-lyases that contain solvent-exposed [4Fe–4S]^2+^ clusters [24]. Recovery of these metal clusters requires reduction which is thought to be done by ferredoxin [25]. Thus, during oxidative stress, induction of *Xac*-FNR might be important for providing reduced ferredoxin. Another unwanted situation is the build-up of NADPH levels, which may favor the propagation of active oxygen species through the reduction of Fe^3+^ [9]. It is likely that FNR acts through NADPH oxidation using any electron acceptor that is available and maintains NADPH at tolerable levels during oxidative stress conditions.

### Expression analysis of Xac-FNR under oxidative stress conditions

**Figure 3:**
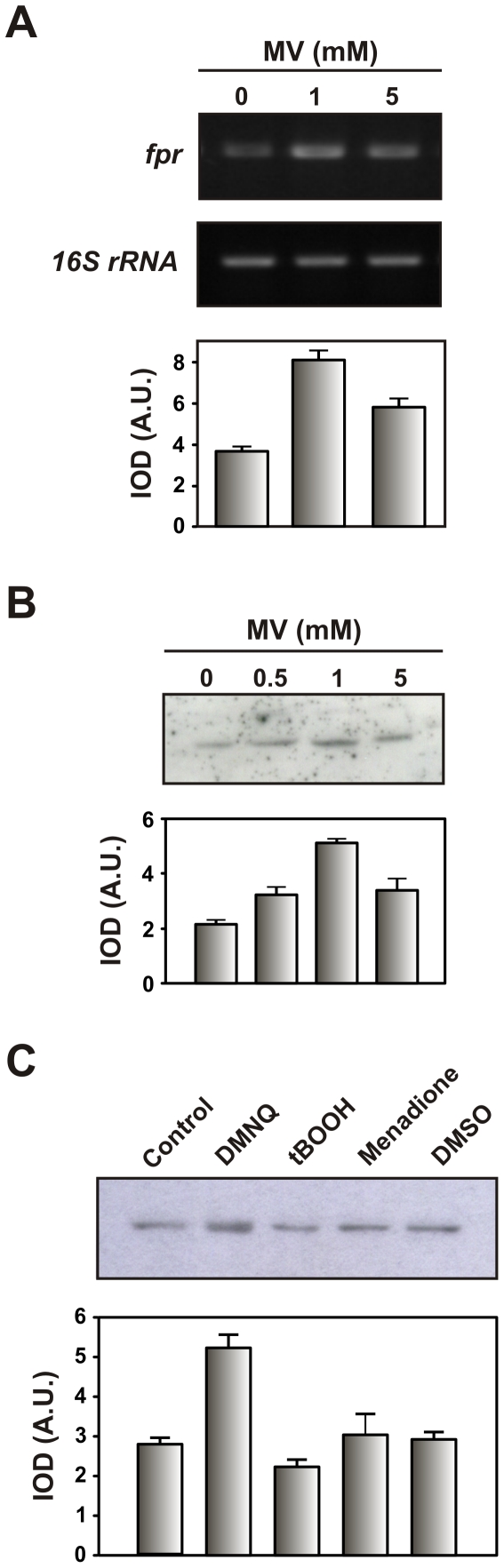
Expression analysis of *Xac*-FNR in response to oxidative treatments. (A) Amplified products of the *Xac fpr* gene by semiquantitative RT-PCR using RNA preparations from *Xanthomonas axonopodis* pv. citri cultures grown in SB medium to the early exponential phase and exposed to the indicated concentrations of MV for 15 min. 16S rRNA was used as a loading control and for the quantitation of the total RNA in the RT-PCR experiments. (B) *Xac*-FNR accumulation after MV-dependent induction. Cleared extracts correspond to 25 µg of total soluble protein. Samples were analyzed by SDS-PAGE and immunoblot analysis using specific antisera. (C) Effect of 2,3-dimetoxy-1,4-naphthoquinone (DMNQ) 500 µM, tert-butyl hydroperoxide (tBOOH) 500 µM, menadione 100 µM and dimethyl sulfoxide (DMSO) on *Xac*-FNR protein expression. Cleared extracts correspond to 30 µg of total soluble protein and were analyzed by Western blot. The graphs below the gels in (A), (B) and (C) show the expression profiles that were obtained by densitometric quantification of the band intensities. Experiments were performed in triplicate with similar results, and the error bars indicate ±1 standard deviation of the mean (IOD, integrated optical density; A.U., arbitrary units).

In order to investigate the involvement of *Xac*-FNR in the oxidative stress response in *X. axonopodis* pv. citri, *fpr* expression at the mRNA level was assessed using semiquantitative RT-PCR analysis. As shown in Figure 3A, expression of *fpr* was detected in normal growth conditions and exhibited a 2.2-fold induction in the presence of 1 mM MV. However, exposure to 5 mM MV led to less of an increased induction of the gene (1.5-fold). Analysis of the *Xac*-FNR protein abundance by Western blot analysis was highly correlated with the expression pattern obtained by RT-PCR (Figure 3B).

*Xac*-FNR was also induced by exposure to the superoxide-generating agent 2,3-dimethoxy-1,4-naphthoquinone (DMNQ, 500 µM) to the same extent as MV 1 mM (Figure 3C). Induction of the *fpr* gene by superoxide-generating agents was previously reported in *E. coli* as a member of the SoxRS regulon [26] and in *Pseudomonas putida* under the control of FinR, a redox-sensing transcriptional regulator [27]. In *E. coli* the SoxRS regulon is also activated by hydrogen peroxide [28]. In *X. axonopodis* pv. citri SoxR was identified [29] but there is no evidence concerning the inclusion of *Xac*-FNR in this regulon. Nevertheless, the induction of *fpr* that was observed after exposure to superoxide-generating agents in *X. axonopodis* pv. citri suggests that FNR could serve a protective role against oxidative stress in this bacterium.

### Structural and kinetic analyses

**Figure 4:**
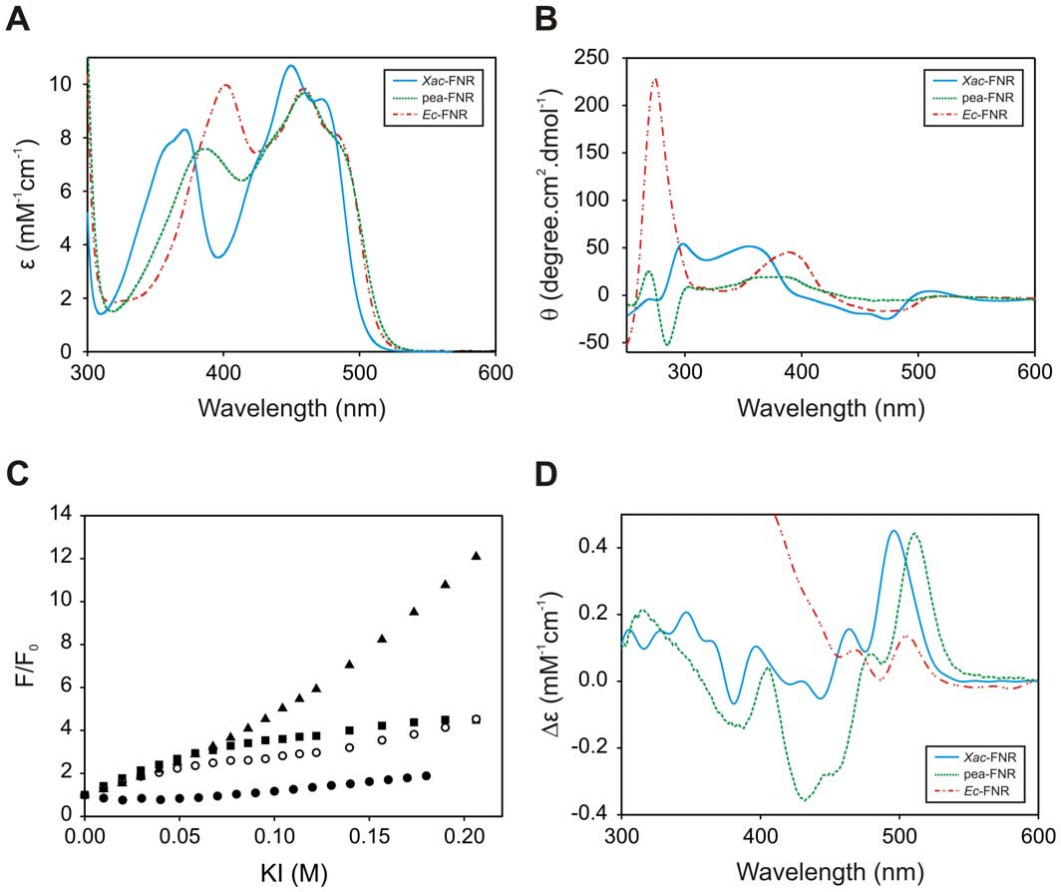
Spectroscopic analyses of *Xac*-FNR and comparison with the pea and *E. coli* enzymes. (A) UV-visible spectra displayed by the different plant-type FNRs. (B) Near-UV and visible CD spectra of the enzymes. (C) FAD solvent accessibility studied by quenching with KI. *Xac*-FNR (○), pea-FNR (▪), *Ec*-FNR (•), free FAD (▴). (D) Differential UV-visible spectra elicited by the interaction between the enzymes and NADP^+^. The spectra were obtained by the subtraction of the spectra of FNR in the presence of 0.3 mM NADP^+^ and the free enzyme. Different colors were employed for each FNR variant: cyan, *Xac*-FNR; green, pea-FNR; and red, *Ec*-FNR.

The UV-visible absorption and CD spectra were collected in order to obtain information about the FAD isoalloxazine environment. Figure 4A shows the UV-visible spectrum of *Xac*-FNR, and the representative spectra of plastidic and subclass II bacterial FNRs (pea and *E. coli*, respectively) for comparison. The UV-visible spectrum of *Xac*-FNR showed the typical pattern observed for subclass I bacterial FNRs with maxima at 450 nm and 372 nm [30]. Maxima for *Xac*-FNR were detected at lower wavelengths with respect to pea-FNR and *Ec*-FNR. In plastidic and subclass II FNRs, a tyrosine is stacked on the *re*-face of the FAD isoalloxazine, which stabilizes the prosthetic group through an aromatic interaction. The *Xac*-FNR contains an alanine that faces the isoalloxazine. Thus, the absence of this stabilizing aromatic interaction may increase the energetic levels of the isoalloxazine electronic transitions and result in the observed blue-shift spectral change [31].

The CD spectra of *Xac*-FNR displayed the same spectral trend that was observed for subclass I bacterial FNR from *Rhodobacter capsulatus* (Figure 4B) [32] except in the near UV region. Previous studies have demonstrated that this CD spectral region is susceptible to the polarity of the solvent [33]. The maximum observed at 270 nm in the *R. capsulatus* FNR CD spectrum was not detected in *Xac*-FNR. Therefore, in spite of the high structural homology between these enzymes, there are differences in the isoalloxazine environment between both members of the subclass I FNR. These differences may be related to the lengths of the carboxy-terminal region, which is shorter in *Xac*-FNR compared to *R. capsulatus* FNR [34] (Figure 1). The shortened carboxy-terminus may allow for the FAD isoalloxazine to be more accessible to the solvent.

Measurement of FNR fluorescence quenching by titration with a dynamic quencher can be used to analyze the accessibility of FAD [35], [36]. Using this experimental approach, we detected that the FAD isoalloxazine was more exposed to the solvent in *Xac*-FNR and in pea-FNR than in *Ec*-FNR (Figure 4C).

It has been previously observed that the intensity of the peak at 515 nm in the differential UV-visible spectra of FNR elicited by NADP^+^ was proportional to the extent of NADP^+^ nicotinamide stacking on the isoalloxazine [37]. The intensity of the signal obtained with *Xac*-FNR was similar to the plastidic FNR, and it was higher than the signal obtained with *Ec*-FNR (Figure 4D) and *R. capsulatus* FNR [38]. The *K*_d_ value for the *Xac*-FNR-NADP^+^ complex was lower than those reported for *R. capsulatus* FNR (9.7 µM vs. 222 µM, Table 1, Figure S1 and [38]) and similar to that of the plastidic type FNRs (Table 1 and [20]). Consequently, in *Xac*-FNR the productive binding of NADP(H) was improved compared to other bacterial subclass I enzymes. These results indicate that in *Xac*-FNR when the NADP is bound to the enzyme, the catalytic competent conformation of the nucleotide is enhanced, resulting in a more efficient enzyme.

**Table 1:** Kinetic parametersa of NADPH and NADH diaphorase reactions that were catalyzed by Xac-FNR, pea-FNR and Ec-FNR, and the dissociation constants for the different complexes with NADP+ b.

	NADPH				NADH			
FNR	*K*_m_ (µM)	*k*_cat_ (s^−1^)	*k*_cat_/*K*_m_ (µM^−1^s^−1^)	*K*_d_ (µM)	*K*_m_ (mM)	*k*_cat_ (s^−1^)	*k*_cat_/*K*_m_ (µM^−1^s^−1^)	NADPH/NADH specificity
*Xac*-FNR	10.8±0.5	121.9 ±− 1.8	11.3	9.7±0.4	3.3±1.4	3.2±1.3	0.001	1600
pea-FNR	15.3±4.3 c	374.3±18 c	24.5 c	10.9±3.1^c^	14.3±3.9 c	7.0±2.1 c	0.0005 c	49060 c
*Ec*-FNR	8.3±1.3 c	38.2±3.5 c	4.6 c	5.9±0.6 c	<0.05 d	Nd e	Nd e	Nd e

a Each parameter value represents the average of three independent determinations. A description of the calculation methods that were employed is reported in the Materials and Methods. The original data are depicted in Figure S2.

b Potassium ferricyanide reduction was assessed using the diaphorase assay of Zanetti [41] in 50 mM Tris-HCl (pH 8.0) using NADPH or NADH as the substrate.

c Values of parameters for pea-FNR and *Ec*-FNR were obtained from a reference [49].

d An estimate of the limit of the determination based on the tested sensitivity of the method.

e Not determined.

FNRs display strong preference for NADP(H) and are very poor NAD(H) oxidoreductases (Table 1, Figure S2 and [20]). In contrast, various redox compounds, including complexed metals and aromatic molecules, can operate as mono and bi-electronic acceptors *in vitro*, in the so-called diaphorase reaction [39]. *Xac*-FNR showed higher NADPH-diaphorase activity compared to *R. capsulatus* FNR (121.9 s-^1^ vs. 7.2 s^−1^, Table 1 and [32]) and *Ec*-FNR (Table 1). In addition, *Xac*-FNR showed higher NADH-diaphorase activity than *Ec*-FNR (Table 1). An increase in the interaction between the nicotinamide and the isoalloxazine has been postulated to be the cause for the decrease in the discrimination of substrate in FNR proteins [37]. Therefore, our results indicate that in *Xac*-FNR a greater interaction between the isoalloxazine and NADP(H) nicotinamide occurs when compared to other FNR proteins from bacterial subclass I. This increased interaction may have some functional relevance.

### Profile of thermal denaturation

**Figure 5:**
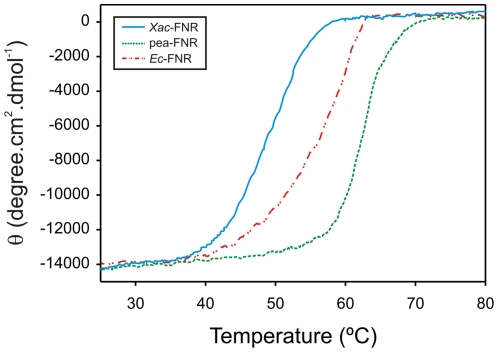
Thermal stability of the different plant-type FNRs according to the folding-unfolding transitions that were observed for the enzymes. Cyan, *Xac*-FNR; green, pea-FNR; and red, *Ec*-FNR.

Figure 5 shows the thermal unfolding curves for the different FNR variants, where *Xac*-FNR has the lowest thermal stability. However, the low stability does not impede *Xac*-FNR to perform its biological function during the life cycle of the bacteria. The optimal growth temperature of *X. axonopodis* pv. citri is 28°C [5], which is lower than the melting temperature of *Xac*-FNR (Table 2 and Figure S3). The absence of an aromatic residue that stacks against the FAD isoalloxazine may contribute to the decreased stability of *Xac*-FNR. Site-directed mutagenesis studies have revealed that the replacement of the carboxy-terminal tyrosine in pea-FNR to a serine induced a 2.6 kcal/mol destabilization [40]. The lack of an aromatic residue that faces the FAD isoalloxazine in *Xac*-FNR allows for an improved interaction of the nicotinamide portion of NADP(H) with the prosthetic group; however, it could be the cause of the low thermal stability of the protein.

**Table 2:** Kinetic parameters a for cytochrome c reductase with pea ferredoxin, E. coli flavodoxin, and E. coli ferredoxin, and the melting temperatures of thermal unfolding transitions for Xac-FNR, pea-FNR and Ec-FNR b.

	pea ferredoxin			E. coli ferredoxin			E. coli flavodoxin			
FNR	K_m_ (µM)	k_cat_ (s^−1^)	k_cat_/K_m_ (µM^−1^s^−1^)	K_m_ (µM)	k_cat_ (s^−1^)	k_cat_/K_m_ (µM^−1^s^−1^)	K_m_ (µM)	k_cat_ (s^−1^)	k_cat_/K_m_ (µM^−1^s^−1^)	T_M_ (°C) c
Xac-FNR	<0.1 d	<0.1 d	Nd e	<0.1 d	<0.1 d	Nd e	<0.1 d	<0.1 d	Nd e	48.1±0.8
pea-FNR	2.2±0.2 f	75.0±0.5 f	34.1 f	Nd e	Nd e	Nd e	Nd e	Nd e	Nd e	61.5±0.5
Ec-FNR	1.4±0.1 f	22.8±0.2 f	16.3 f	0.074±0.023 f	12.3±1.2 f	164.9 f	2.3±0.2 f	8.9±1.0 f	3.9 f	57.5±0.7

a Each value represents the average of three independent determinations. A description of the calculation methods that were employed and the activity determinations are reported in the Materials and Methods. The original data are depicted in Figure S3.

b Cytochrome *c* reduction was determined at 550 nm (ε_550_ = 19 mM^−1^ cm^−1^) as described in the Materials and Methods.

c T_M_ is the temperature of the midpoint of the thermal denaturation transition and was determined as described in the Materials and Methods.

d An estimate of the limit of the determination based on the tested sensitivity of the method.

e Not determined.

f Values of parameters for pea-FNR and *Ec*-FNR were obtained from a reference [49].

### Analysis of the redox partner of Xac-FNR

Ferredoxins and flavodoxins are considered the main redox-partners of FNRs [18], [20]. The electron transfer from NADPH to ferredoxin catalyzed by FNRs can be followed using cytochrome *c* as final electron acceptor in a coupled assay known as cytochrome *c* reductase activity [41]. Reduction of cytochrome *c* shows a strict requirement for ferredoxin. The reaction is most often described as consisting of two hemi-reactions: FNR-catalyzed reduction of ferredoxin by NADPH, and the subsequent reoxidation of the iron-sulfur protein by cytochrome *c*. *Xac*-FNR was not able to reduce ferredoxin or flavodoxin from *E. coli* or pea ferredoxin. Expected activity values were obtained in parallel experiments with the plastidic and *E. coli* enzymes (Table 2).

**Figure 6:**
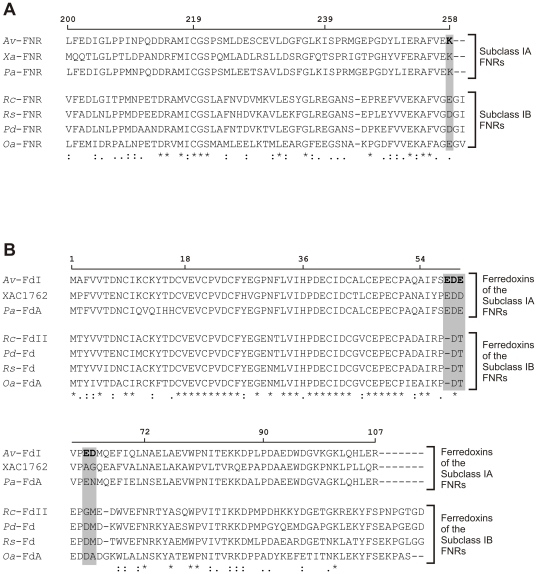
Analysis of *Xac*-FNR's redox partner by homology sequence. (A) Alignment of primary structures of subclass I bacterial FNRs from *A. vinelandii* (*Av*-FNR, gb: YP_002800963.1), *X. axonopodis* pv. citri (*Xa*-FNR, gb: NP_641792.1), *Pseudomonas aeruginosa* (*Pa*-FNR, gb: YP_001347117.1), *R. capsulatus* (*Rc*-FNR, gb: ADE85336.1), *Rhodobacter sphaeroides* (*Rs*-FNR, gb: YP_002524612.1), *Paracoccus denitrificans* (*Pd*-FNR, gb: ABL68770.1) and *Oceanicaulis alexandrii* (*Oa*-FNR, gb: ZP_00952506.1). Sequence regions from amino acid 200 to the carboxy-terminus are shown. In bold is the amino acid from *Av*-FNR that is involved in the interaction with ferredoxin I, as was previously reported [42]. (B) Alignment of ferredoxin I from *A. vinelandii* (*Av*-FdI, gb: AAA22125.1) and ferredoxins from *X. axonopodis* pv. citri (XAC1762, gb: NP_642090.1), *P. aeruginosa* (ferredoxin A, *Pa*-FdA, gb: AAF89693.1), *R. capsulatus* (ferredoxin II, *Rc*-FdII, gb: YP_003578927.1), *P. denitrificans* (*Pd*-Fd, gb: ABL69923.1), *Rhodobacter* sp. (*Rs*-Fd, gb: ZP_05844833.1) and *O. alexandrii* (ferredoxin A, *Oa*-FdA, gb: ZP_00953239.1). In bold is the peptide involved in the interaction with *Av*-FNR identified by cross-linking experiments, as was previously reported [42]. Potential residues of *Av*-FdI that interact with Lys258 of *Av*-FNR are shaded in gray. Numbers over the sequences correspond to the *A. vinelandii* proteins. The alignments were performed using ClustalX 2.0.11.

**Figure 7:**
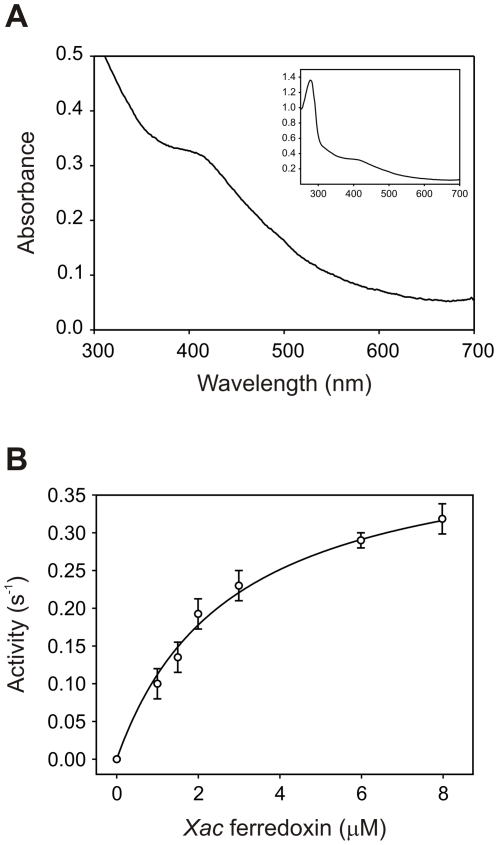
Characterization of ferredoxin XAC1762. (A) UV-visible spectrum displayed by ferredoxin XAC1762. (B) Kinetics of the cytochrome *c* reductase reaction of *Xac*-FNR with ferredoxin XAC1762 as substrate.

The analysis of the *X. axonopodis* pv. citri genome showed the existence of five putative ferredoxins and one flavodoxin [29]. Interestingly, the ferredoxin XAC1762 showed 68% identity and 80% similarity to ferredoxin I from *A. vinelandii*, which has been demonstrated to interact productively with FNR in this bacterium [42]. Taking into account the high similarity between *Xac*-FNR and the FNR from *A. vinelandii* (Figure 6A and Figure S4), we postulated ferredoxin XAC1762 as a potential redox partner of *Xac*-FNR. To test this hypothesis, we cloned, expressed and purified the ferredoxin coded by the XAC1762 sequence. The purified protein displayed a typical broad UV-visible spectrum with a band at 407 nm, which is indicative of [4Fe-4S] or [3Fe-4S] ferredoxins [43] (Figure 7A). The NADPH-cytochrome *c* reductase activity of *Xac*-FNR with different amounts of XAC1762 ferredoxin was measured under argon (Figure 7B) and a *K*_m_ value of 2.8 µM and a *k*_cat_ of 0.42 s^−1^ were obtained. These results indicate that XAC1762 ferredoxin is one of the possible redox partners of *Xac*-FNR. The activity observed with *Xac*-FNR and the ferredoxin XAC1762 is lower than the corresponding value obtained for the *E. coli* couple, although *Xac*-FNR displayed higher diaphorase activity.

Crosslinking experiments between FNR and ferredoxin I from *A. vinelandii* were employed to determine the important residues for complex formation [42]. Lys258 from FNR and an acidic patch formed by Glu57-Asp58-Glu59/Glu62-Asp63 of the above mentioned ferredoxin were identified. *Xac*-FNR and ferredoxin XAC1762 from *X. axonopodis* pv. citri contain homologous residues at equivalent positions (see Figure 6A and B). Thus, it may be suggested that *Xac*-FNR and ferredoxin XAC1762 from *X. axonopodis* pv. citri contain the structural features necessary for this interaction. Lys258 is not conserved in all subclass I bacterial FNRs and is replaced in some of them by a glutamate in the equivalent position as occurs in *R. capsulatus* [21] (Figure 6A). Consequently, it can be suggested that the FNRs may have evolved in order to acquire structural features on the carboxy-terminal region that allow for the modulation of the specificity of the interaction with their redox partners. We suggest that subclass I bacterial FNRs be further subdivided into two new groups: subclass IA, which the prototype would be the FNR from *A. vinelandii*; and subclass IB, which the representative member would be the FNR from *R. capsulatus*. The main differences between both subclasses are located at the carboxy-terminal region. While enzymes from subclass IA have Lys258 (numbering for *A. vinelandii*), the subclass IB FNR proteins contain a glutamate or an aspartate at the equivalent position and a longer carboxy-terminal region (Figure 1 and Figure 6A). We searched for all proteins with carboxy-terminal regions that were similar to FNR from *R. capsulatus* in the Data Bank. We found that the FNRs from *Rhodobacter sp.*, *Paracoccus denitrificans* and *Oceanicaulis alexandrii* meet these criteria (Figure 6A). Consequently, subclass IA is defined by the carboxy terminal sequence VEK and the subclass IB by the sequence (V/A)G(E/D)G(I/V).We analyzed the ferredoxins that may function as substrates for these enzymes. In all cases, the ferredoxins that might act as redox partners of subclass IA FNRs contain the Glu-Asp-Glu triad and the acidic patches while those of subclass IB FNRs displayed conserved Asp-Thr-Glu and basic amino acids at positions 56 and 73/74 (Figure 6B).

In our work, we demonstrated that *Xac*-FNR was regulated by the accumulation of reactive oxygen species, and that this protein is able to substitute the endogenous *E. coli* FNR in its antioxidant role. Purified ferredoxin XAC1762 was shown to be one of the possible substrates of *Xac*-FNR. Reduction of this ferredoxin by *Xac*-FNR may contribute to the oxidative stress response in *X axonopodis* pv. citri, by promoting a decrease in the intracellular NADPH levels. Structural and functional analyses of *Xac*-FNR suggests that the bacterial subclass I can be further classified into subgroups IA and IB. Subclass IA bacterial FNRs (to which *Xac*-FNR belongs) may interact with ferredoxins similar to ferredoxin I of *A. vinelandii* and ferredoxin XAC1762 of *X. axonopodis* pv. citri.

## Materials and Methods

### Bacterial strains, plasmids and growth conditions

Bacterial strains and plasmids used in this study are described in Table 3. *X. axonopodis* pv. citri cells were grown aerobically in Silva Buddenhagen (SB) medium (5 g l^−1^ sucrose, 5 g l^−1^ yeast extract, 5 g l^−1^ peptone, and 1 g l^−1^ glutamic acid at pH 7.0) at 28°C with shaking at 200 rpm. *E. coli* strains were grown at 37°C in Luria-Bertani (LB) or M9 minimal media that was supplemented with 0.2% (w/v) glucose [44]. IPTG was added to a final concentration of 0.5 mM when the expression of plasmid-borne genes was desired. Antibiotics were added to the media at the following final concentrations: ampicillin (Ap), 25 µg ml^−1^ for *X. axonopodis* pv. citri and 100 µg ml^−1^ for *E. coli*; kanamycin (Km), 40 µg ml^−1^; tetracycline (Tc), 15 µg ml^−1^ and chloramphenicol (Cm), 30 µg ml^−1^. The *X. axonopodis* pv. citri strain Xcc99–1330 was kindly provided by Blanca I. Canteros (INTA; Bella Vista, Argentina).

**Table 3:** Bacterial strains and plasmids used in this study.

Strain/plasmid	Genotype or relevant characteristics	Source/reference
**Strains**		
*Xanthomonas axonopodis* pv. *citri*		
Xcc99-1330	Wild type, Ap^r^	B. I. Canteros
*Escherichia coli*		
JM109	*HsdR17 endA1 Recal thi gyrA96 relA1 recA1 supE44 λ^-^Δ (lac-proAB),* [F', traD36, proA^+^B^+^, *lacI^q^ZΔM15*]	[44]
GC4468	F^-^ Δ*lac* U169 *rpsL*	[54]
RR6A	GC4468 *fpr*, Km^r^	[9]
BL21(DE3)pLysS	F- *ompT hsdS*_B_ (r^-^_B_m^-^_B_) *dcm gal* (DE3) pLysS, Cm^r^	Novagen
C41(DE3)	F- *ompT hsdS*_B_ (r^-^_B_m^-^_B_) *dcm gal* (DE3)	[55]
**Plasmids**		
pGEM-T Easy	Vector for cloning PCR products, Ap^r^	Promega
pUC119	pBR322 derivative, *lacZ*, Ap^r^	[45]
pEE1010	pUC18 carrying the *E. coli fpr* gene, Ap^r^	[56]
pRKISC	pRK415 vector carrying an ISC operon, Tc^r^	[47]
pUC/*Xac*FNR	pUC119 carrying the *Xac fpr* gene, Ap^r^	This study
pET/*Xac*FNR	pET28a carrying the *Xac fpr* gene, Km^r^	This study
pET/*Xac*Fd	pET28a carrying the XAC1762 gene, Km^r^	This study

Ap, ampicillin; Km, kanamycin; Cm, chloramphenicol; Tc, tetracycline.

### Complementation of the E. coli fpr-null mutant with Xac-FNR

The coding sequence for the *Xac*-FNR was amplified by PCR using oligonucleotides FPR-F2 (5'-TTCCAAGCTTCATGTCTTCCGCTTTTGGCGC-3') and FPR-R (5'-TTCCGAATTCGCGCGTCACTTTTCGACGAA-3') as primers, and the *X. axonopodis* pv. citri genomic DNA was used as the template. The PCR product (806 bp) was cloned into the pGEM-T Easy plasmid (Promega), digested with *Hind*III and *Eco*RI, and ligated to compatible sites in pUC119 [45]. After sequencing the resulting plasmid, pUC/*Xac*FNR was transformed into *E. coli* strain RR6A (*fpr*-null mutant) [9]. Expression of the recombinant protein in soluble cell extracts was verified by SDS-PAGE, and immunoblot analysis was performed with specific antisera.

### Bacterial viability assay

Bacterial resistance to MV was evaluated by the disk diffusion method. Briefly, 100 µl of a bacterial suspension that contained ∼10^9^ cells ml^−1^ was mixed with 3 ml 0.7% (w/v) molten agar at 42°C and was poured onto M9-agar plates supplemented with the corresponding antibiotics and 0.5 mM IPTG when required. After hardening, 5 µl of a 100 mM MV solution was added onto paper disks (5-mm diameter) placed on the agar surface. The zones of growth inhibition were measured after incubation for 24 h at 37°C.

### Determination of Xac-FNR expression

*X. axonopodis* pv. citri overnight cultures were diluted into fresh SB medium with 2% inoculum. Bacterial suspensions were grown at 28°C to OD600 0.5–0.7 (exponential phase) and were incubated with the oxidative agents for 15 min. For cell extract preparation, the cultures were harvested by centrifugation at 10000 g for 10 min at 4°C. Bacteria were washed and resuspended in 500 µl of ice-cold potassium phosphate buffer (50 mM; pH 7.0) that contained 1 mM PMSF and were disrupted by intermittent sonication. The suspensions were clarified by centrifugation at 12000 g for 20 min at 4°C. Protein concentrations in the soluble cell extracts were determined using a dye-binding assay [46] that used bovine serum albumin as a standard. The soluble fractions were resolved by SDS-PAGE and transferred to nitrocellulose membranes, and FNR was detected with specific antisera using secondary antibodies that were conjugated to alkaline phosphatase. Immunoreactive bands were integrated using Gel-Pro Analyzer Software 3.1 (Media Cybernetics).

### RNA extraction and semiquantitative reverse transcription PCR (RT-PCR)

The total RNA from *X. axonopodis* pv. citri cells was isolated using TRIzol^®^ (Invitrogen) according to the manufacturer's instructions. After extraction, the RNA was treated with RNase-free DNase (Promega), and its integrity was determined by agarose gel electrophoresis. Semiquantitative analyses of *fpr* transcript levels were performed using a two-step RT-PCR approach that employed the primers fprRT-F (ATGTCTTCCGCTTTTGGCGC) and fprRT-R (CTGGGTGAGGATCACCTTGT). For cDNA synthesis, total RNA (1 µg) was added to 20 µl of a reverse transcription reaction that contained 4 µl 5× M-MLV buffer (Promega), 0.5 mM dNTP mixture, 0.5 µg gene-specific primer, and 200 U M-MLV reverse transcriptase (Promega), and the reaction was incubated for 60 min at 42°C. Reverse transcription was terminated by incubation at 94°C for 5 min. Control reactions, where RT was omitted, were performed in parallel for all the samples to rule out the possibility of amplification from contaminating DNA. PCR reactions were performed with 2 µl of cDNA template under the following conditions: 25 cycles of denaturation at 94°C for 1 min, annealing at 65°C for 1 min, and extension at 72°C for 1 min; and a final extension step at 72°C for 5 min. The number of cycles, which avoided reaching the plateau of PCR, was previously determined by taking samples at different cycle numbers during PCR amplification and analyzing the products obtained by agarose gel electrophoresis. As a control, a 217-bp fragment of 16S rRNA was amplified using primers 16S-F (TGGTAGTCCACGCCCTAAACG) and 16S-R (CTGGAAAGTTCCGTGGATGTC) and the same PCR conditions; however, only 1% of the cDNA synthesis reaction was used as the template due to the high abundance of 16S rRNA in the total RNA extracts. RT-PCR products were resolved on 1.5% (w/v) agarose gels, and the gels were densitometrically quantified using Gel-Pro Analyzer Software 3.1 (Media Cybernetics).

### Preparation of recombinant proteins

Recombinant *Xac*-FNR was obtained by expression in *E. coli*. Briefly, a pET/*Xac*FNR expression vector was constructed by inserting the coding sequence of *Xac*-FNR into the pET28a vector (Novagen). The coding sequence for *Xac*-FNR was amplified using PCR with the primers FPR-F (TATCTCTCCATATGTCTTCCGCTTTTGGCGC) and FPR-R (TTCCGAATTCGCGCGTCACTTTTCGACGAA), and the *X. axonopodis* pv. citri genomic DNA was used as the template. To facilitate the cloning process, the *Nde*I and *Eco*RI restriction sites were introduced in the primers FPR-F and FPR-R, respectively. The PCR product (806 bp) was cloned into the pGEM-T Easy plasmid (Promega), digested with *Nde*I and *Eco*RI and ligated into compatible sites in pET28a. The plasmid pET/*Xac*FNR contained the entire *Xac*-FNR coding region fused in-frame to an N-terminal hexahistidine tag. For expression in *E. coli* BL21(DE3)pLysS cells, the cultures were grown at 37°C in LB medium supplemented with kanamycin and chloramphenicol for 3 h and were induced with 0.25 mM IPTG for 6 h at 20°C. *Xac*-FNR was purified by Ni-NTA affinity chromatography and dialyzed against 50 mM Tris-HCl buffer (pH 8.0) in the presence of 150 mM NaCl. The fusion protein was digested with thrombin, and the hexahistidine-tag was removed by another Ni-NTA affinity chromatography procedure.

Ferredoxin XAC1762 were overexpressed in *E. coli* C41 cells transformed with pET/*Xac*Fd and ISC operon expressing plasmid pRKISC [47], as a carboxy-terminal fusion with His6-tag and a TEV recognition site between the His6-tag and the last amino acid of the protein. The pET/*Xac*Fd was constructed by inserting the coding sequence for the ferredoxin XAC1762 into pET28a expression vector. This sequence was amplified by PCR using the primers *Xac*Fd-F (AAGGCCATGGCTTTTGTTGTCACCGAAAACTGC) and *Xac*Fd-R (TGGAAGCTTGCCCTGAAAATACAGGTTTTCGCGCTGCAGCAGCGGCAATTTGTTGGGCTTGCCATCCCATTCGGC) and the *X. axonopodis* pv. citri genomic DNA as a template. To facilitate cloning, the *Nco*I and *Hind*III restriction sites were introduced in the primers *Xac*Fd-F and *Xac*Fd-R, respectively. The PCR product (357 bp) was cloned into the pGEM-T Easy plasmid (Promega), digested with *Nco*I and *Hind*III and ligated into compatible sites in pET28a, rendering pET/*Xac*Fd plasmid. For functional expression, bacteria were grown at 37°C in LB medium supplemented with kanamycin and tetracycline for 3 h and then expression induced by the addition of 0.25 mM IPTG and supplemented with 2 mM ammonium ferric citrate. Then, the cultures were maintained during 16 h at 18°C with mild agitation. *Xac* ferredoxin was purified by Ni-NTA affinity chromatography and dialyzed against 50 mM Tris-HCl buffer (pH 8.0), 150 mM NaCl. The fusion protein was digested with TEV protease and the hexahistidine-tag was removed by another Ni-NTA affinity chromatography procedure.

### Spectral Analyses

UV-visible absorption spectra were recorded on a Shimadzu UV-2450 spectrophotometer. CD spectra were obtained using a JASCO J-810 spectropolarimeter at 25°C. The spectra were recorded in 5.0 µM protein solutions in 0.1 cm path length cuvettes. Fluorescence spectra were monitored using a Varian (Palo Alto, CA) Cary Eclipse fluorescence spectrophotometer that was interfaced with a personal computer. The samples were filtered through G25 Sephadex spin columns that were equilibrated with 50 mM potassium phosphate (pH 8.0) before measurements were collected. The extinction coefficient of *Xac*-FNR was determined by releasing FAD from the protein by treatment with 0.2% (w/v) SDS and quantifying the flavin spectrophotometrically [48].

### Enzymatic Assays

FNR-dependent NADPH-K_3_Fe(CN)_6_ diaphorase activity was determined using previously published methods [41]. The NADH-ferricyanide diaphorase activity was determined in 1 ml reaction medim that contained 50 mM Tris-HCl (pH 8.0), 1 mM potassium ferricyanide, and 1.25–2.5 µM FNR. The cytochrome *c* reductase activity of *Xac*-FNR, using either ferredoxin or flavodoxin, was assayed in reaction medium (1 ml) that contained 50 mM Tris-HCl (pH 8.0), 0.3 mM NADP^+^, 3 mM glucose 6-phosphate, 1 unit of glucose-6-phosphate dehydrogenase, and 50 µM cytochrome *c* [49]. After the addition of approximately 15–100 nM FNR, cytochrome *c* reduction was monitored spectrophotometrically by following absorbance changes at 550 nm (ε_550_ = 19 mM^−1^ cm^−1^). All kinetic experiments were performed at 30°C. In all cases, precautions were taken to ensure the linearity of the enzyme activity, and when appropriate, saturation of the Michaelis-Menten plots was verified.

### Thermal unfolding transitions

Protein stock solutions were diluted to a final concentration of 0.5 µM in 50 mM potassium phosphate (pH 8.0). The CD signal was measured by excitation at 220vnm while the temperature of the sample was increased at a rate of 1°C min^−1^ (from 25 to 80°C). Thermal unfolding transitions were analyzed assuming a two-state approximation, which only the native and unfolded states were significantly populated. The T_M_ was determined by fitting experimental data to the equation, ΔG_(T)_ = ΔH_(TM)_+ΔC_p_(T−T_M_)−T(ΔH_(TM)_/T_M_+ΔC_p_ln(T/T_M_)), as described elsewhere [49], [50].

### Determination of dissociation constants of Xac-FNR complexed with NADP^+^ and protein substrates

The *K*_d_ value of the complex between *Xac*-FNR and NADP^+^ was determined by difference absorption spectroscopy, which was previously described [51]. Briefly, 15 µM flavoprotein in 50 mM Tris-HCl (pH 8.0) was titrated at 25°C with NADP^+^. After each addition, the absorbance spectra (200–600 nm) were monitored. The difference spectra were calculated, and the absorbance differences at the stated wavelengths were plotted against the concentration of NADP^+^. The data were fitted to a theoretical equation for a 1∶1 complex. The sample had been previously filtered through a desalting column that had been equilibrated with 50 mM Tris-HCl (pH 8.0). To determine the *K*_d_ values of the complex between *Xac*-FNR and pea ferredoxin, *E. coli* flavodoxin or *E. coli* ferredoxin, solutions that contained 3 µM enzyme in 50 mM Tris-HCl (pH 8.0) were titrated with the corresponding protein substrate. After each addition, fluorescence quenching at 340 nm (excitation at 270 nm) was determined. Controls were run in parallel to estimate the fluorescence contribution due to the addition of pea ferredoxin, *E. coli* flavodoxin or *E. coli* ferredoxin. The *K*_d_ values were estimated by fitting the fluorescence data to a theoretical equation for a 1∶1 complex [49].

### Determination of parameters

All experimental data were fit to theoretical curves using SigmaPlot (Systat Software Inc., Point Richmond, CA, USA).

### Solvent accessibility of the FAD

Quenching of flavin fluorescence by iodide was used to investigate the relative accessibility of FAD in the FNR variants [52], [53]. The emission fluorescence at 525 nm (λ of emission 450 nm) of a 2 ml sample of FNR in Tris-HCl (pH 8.0) was determined during the titration of KI in cuvettes with a 1-cm path-length at 25°C. The samples were previously filtered through a sephadex G25 column that was equilibrated with 50 mM Tris-HCl (pH 8.0) to separate the free FAD.

## Supporting Information

Figure S1 **Determination of the dissociation constants for the FNR-NADP^+^ complexes.** The absorbance changes elicited by NADP^+^ on each enzyme in the 490–510 nm region were used to calculate the *K*_d_ values by fitting the data to a theoretical equation for a 1∶1 complex. *Xac*-FNR (○), pea-FNR (▪), *Ec*-FNR (•).
(TIF)

Figure S2 **Determination of the kinetic parameters of the diaphorase reaction for the different enzymes.** Kinetics of the ferricyanide reduction by *Xac*-FNR (○), pea-FNR (▪) and *Ec*-FNR (•) using NADPH (A) and NADH (B) as substrates.
(TIF)

Figure S3 **Kinetics of cytochrome** ***c*** **reductase reactions of the different FNR enzymes.** Reduction of cytochrome *c* by pea-FNR (▪) and *Ec*-FNR (•) using pea ferredoxin (A), *E. coli* ferredoxin (B) and *E. coli* flavodoxin (C) as substrates.
(TIF)

Figure S4 **Alignment of the complete sequences of subclass I bacterial FNRs.** Sequences of FNRs from *A. vinelandii* (*Av*-FNR), *X. axonopodis* pv. citri (*Xa*-FNR), *Pseudomonas aeruginosa* (*Pa*-FNR), *R. capsulatus* (*Rc*-FNR), *Rhodobacter sphaeroides* (*Rs*-FNR), *Paracoccus denitrificans* (*Pd*-FNR) and *Oceanicaulis alexandrii* (*Oa*-FNR) were analyzed.
(TIF)
