# Supplementary figures and images for: Structural-Functional Characterization and Physiological Significance of Ferredoxin-NADP+ Reductase from Xanthomonas axonopodis pv. citri
Source: PLoS One. 2011 Nov 9;6(11):e27124. doi: 10.1371/journal.pone.0027124 (PMC3212534; doi:10.1371/journal.pone.0027124)

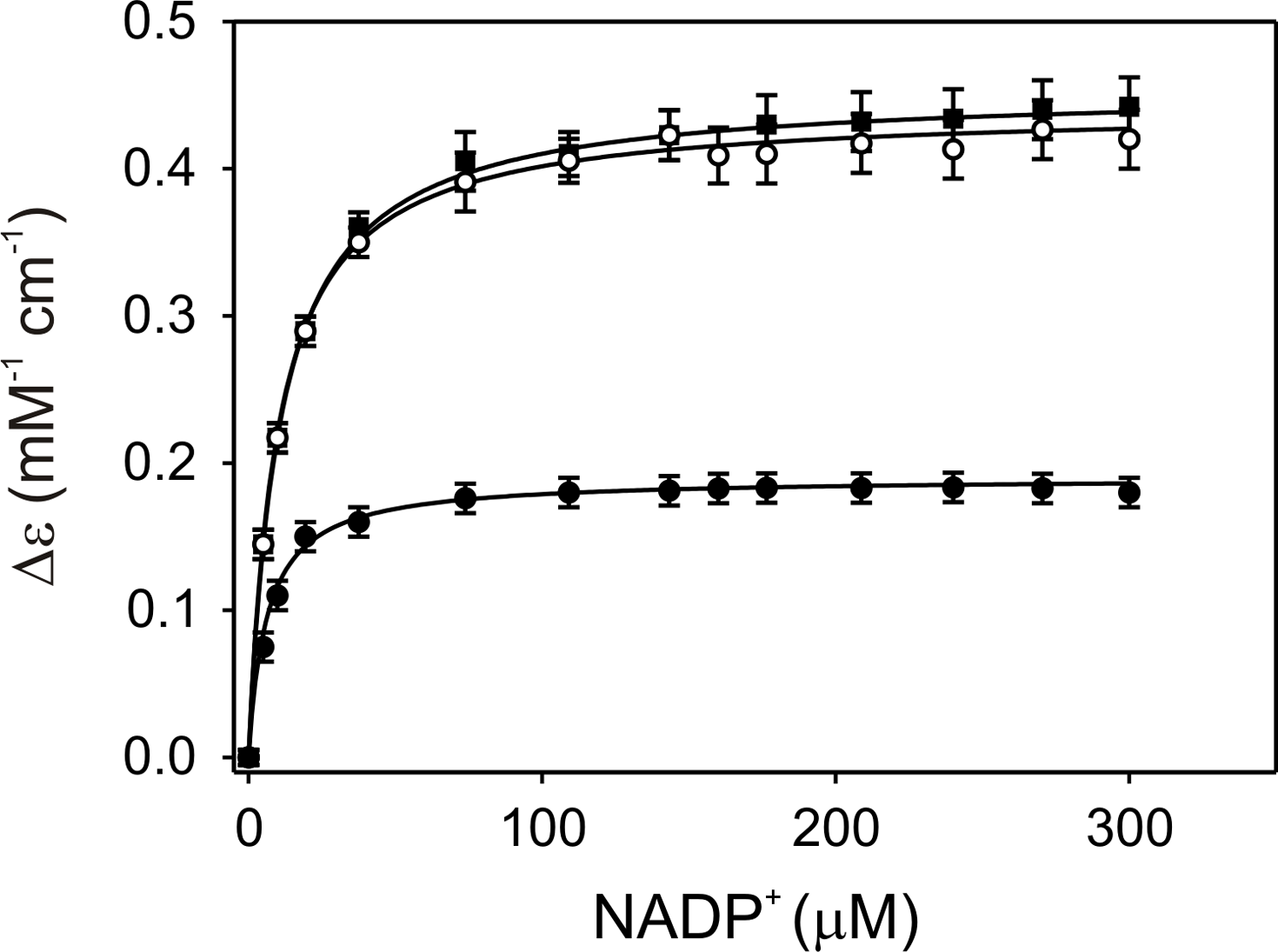

Supplement: Figure S1 — Determination of the dissociation constants for the FNR-NADP+ complexes. The absorbance changes elicited by NADP+ on each enzyme in the 490–510 nm region were used to calculate the K d values by fitting the data to a theoretical equation for a 1∶1 complex. Xac-FNR (○), pea-FNR (▪), Ec-FNR (•). (TIF) [file pone.0027124.s001.tif]

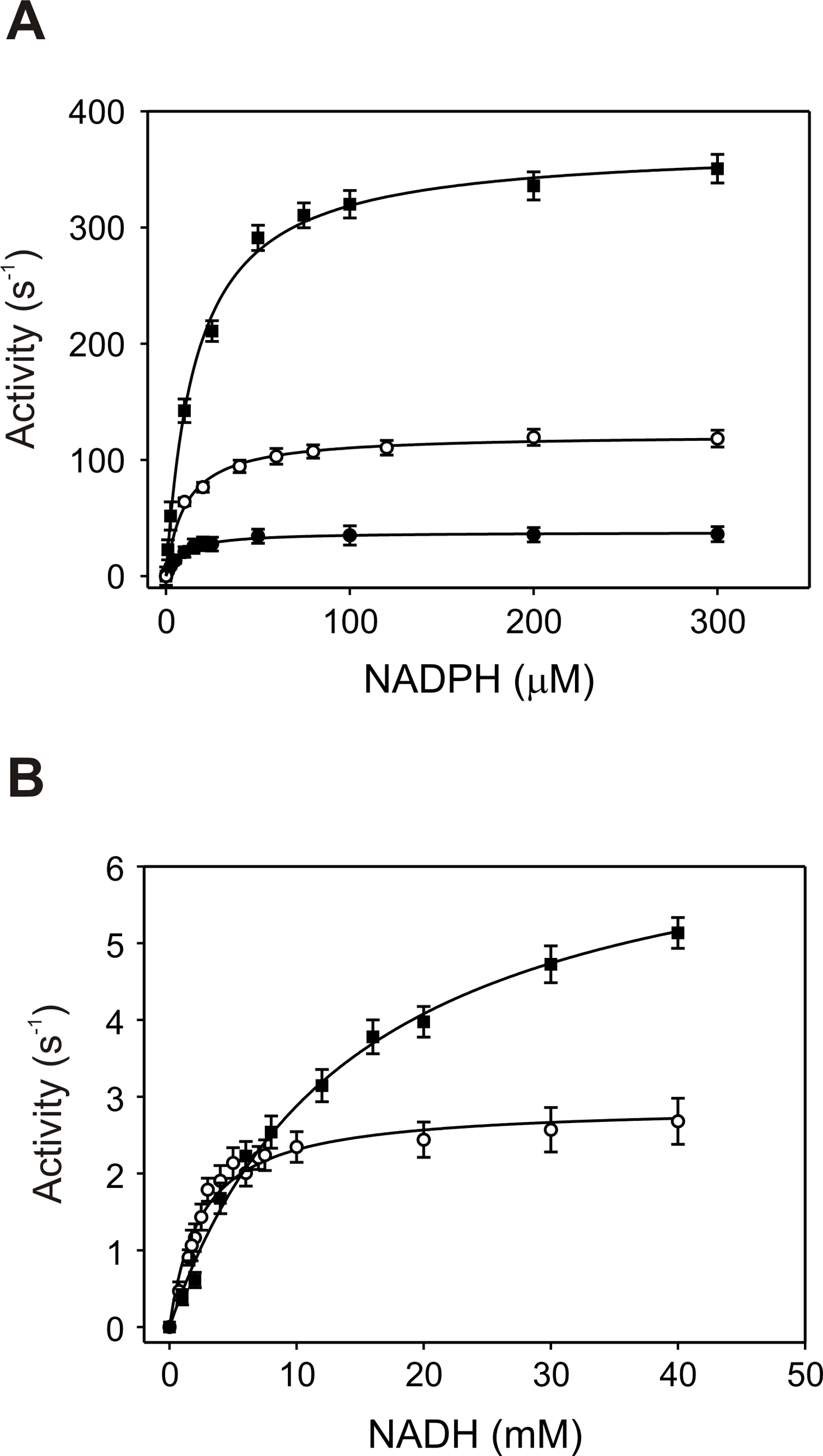

Supplement: Figure S2 — Determination of the kinetic parameters of the diaphorase reaction for the different enzymes. Kinetics of the ferricyanide reduction by Xac-FNR (○), pea-FNR (▪) and Ec-FNR (•) using NADPH (A) and NADH (B) as substrates. (TIF) [file pone.0027124.s002.tif]

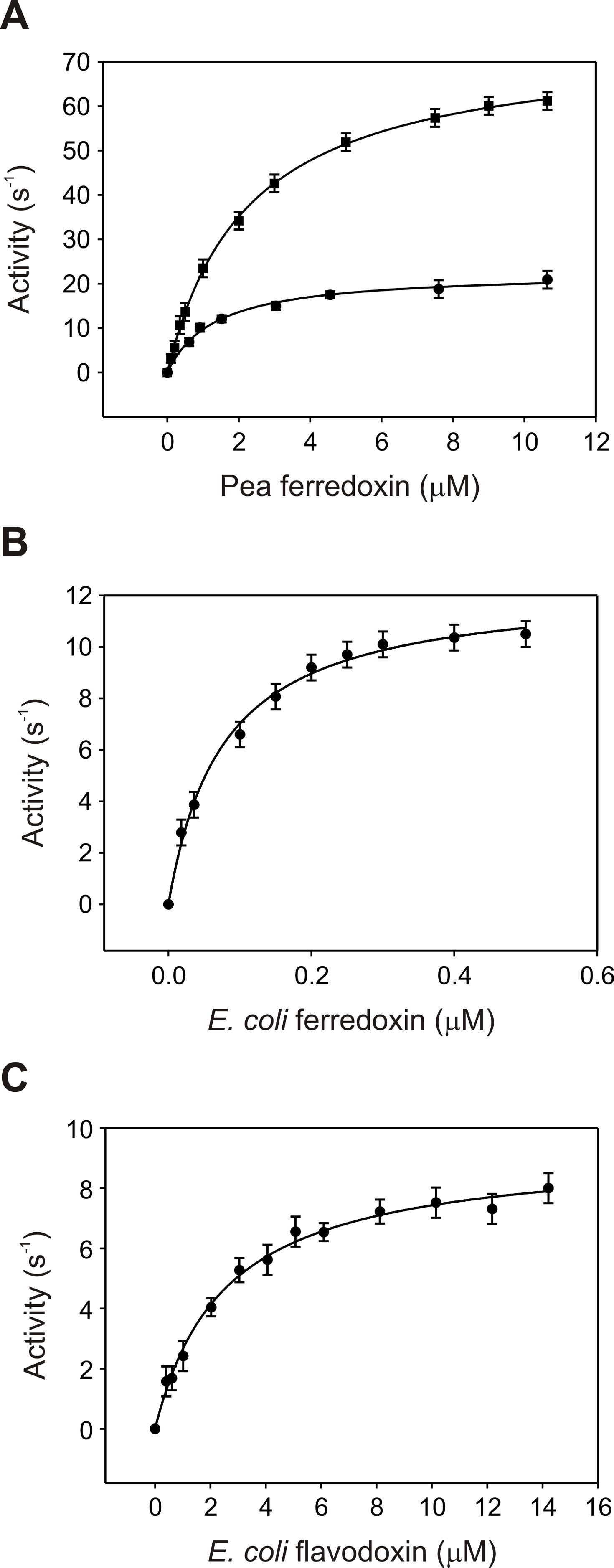

Supplement: Figure S3 — Kinetics of cytochrome c reductase reactions of the different FNR enzymes. Reduction of cytochrome c by pea-FNR (▪) and Ec-FNR (•) using pea ferredoxin (A), E. coli ferredoxin (B) and E. coli flavodoxin (C) as substrates. (TIF) [file pone.0027124.s003.tif]

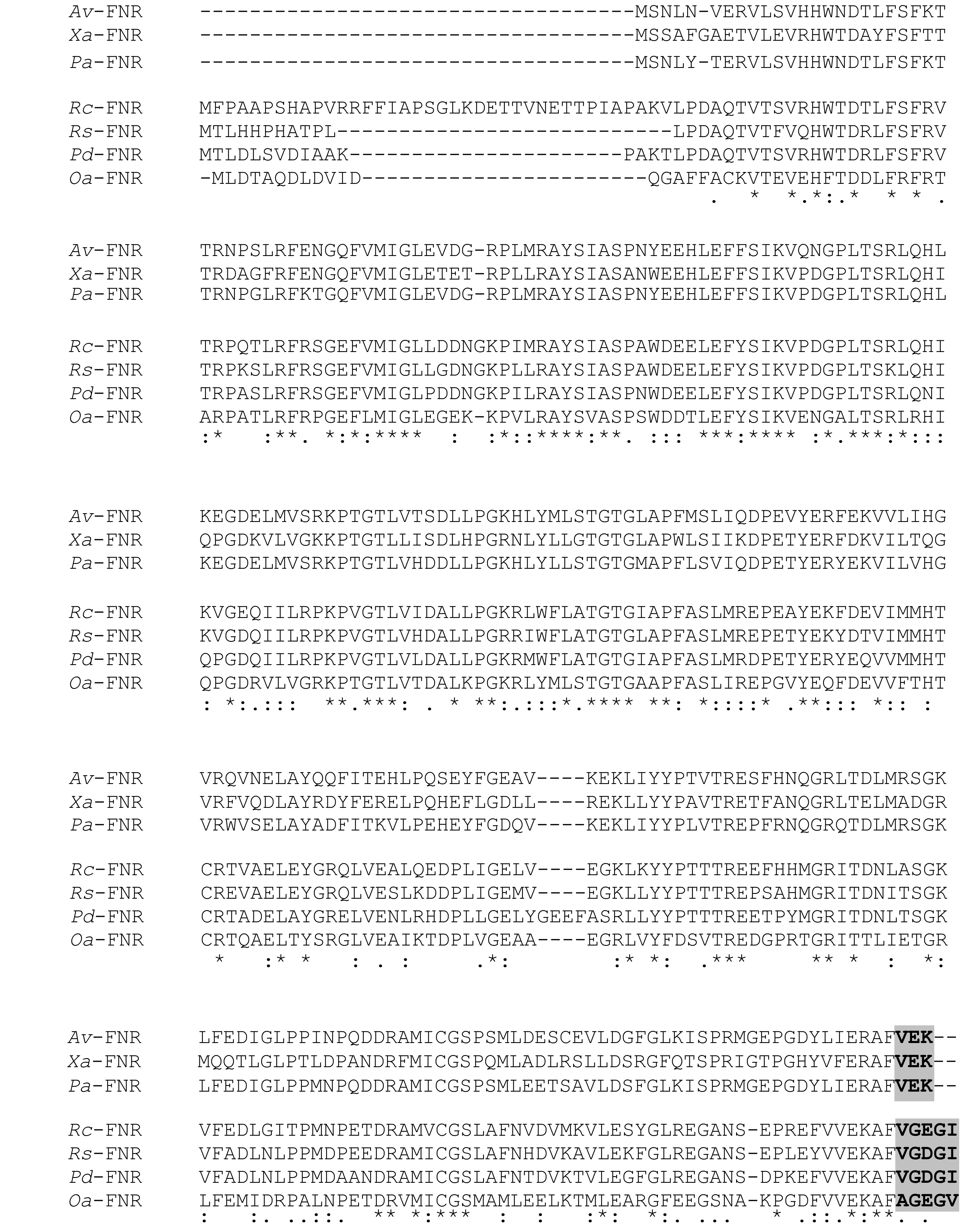

Supplement: Figure S4 — Alignment of the complete sequences of subclass I bacterial FNRs. Sequences of FNRs from A. vinelandii (Av-FNR), X. axonopodis pv. citri (Xa-FNR), Pseudomonas aeruginosa (Pa-FNR), R. capsulatus (Rc-FNR), Rhodobacter sphaeroides (Rs-FNR), Paracoccus denitrificans (Pd-FNR) and Oceanicaulis alexandrii (Oa-FNR) were analyzed. (TIF) [file pone.0027124.s004.tif]
